# Clinical features and early warning factors of symptomatic pulmonary embolism after total joint arthroplasty: a retrospective cohort study

**DOI:** 10.3389/fmed.2026.1915153

**Published:** 2026-09-25

**Authors:** Yanbin Tian, Yun Zhang, Jinguo Ma, Xiaohu Ma, Zhongyong Li, Sheng Li, Qian Liu, Ming Liu, Zeming Yang, Xiaojin Wu

**Affiliations:** 1 Yinchuan Guolong Orthopedic Hospital, Yinchuan, Ningxia, China; 2 Department of Orthopedics, Tongxin County People's Hospital, Wuzhong, Ningxia, China

**Keywords:** clinical features, early recognition model, symptomatic pulmonary embolism, total joint arthroplasty, warning factors

## Abstract

**Background:**

Symptomatic pulmonary embolism (PE) is a severe and life-threatening complication following total joint arthroplasty (TJA). Despite advances in diagnosis, treatment, and prediction, postoperative PE still occurs. This study aimed to investigate the onset time window, initial clinical presentations, and preoperative comorbidity profiles of patients with symptomatic PE after TJA, thereby providing clinical evidence for early identification and perioperative management of post-TJA PE.

**Methods:**

In this retrospective study, patients who underwent TJA at our institution between January 2023 and December 2025 and were diagnosed with postoperative PE via computed tomography pulmonary angiography were assigned to the case group. Non-PE patients were randomly selected at a 1:2 ratio as controls. Data collected included baseline patient characteristics, surgical procedure, time from surgery to PE onset, chief complaints at onset, BMI, and preoperative comorbidities. Age-stratified analyses were conducted to compare symptom distributions. Multivariable logistic regression was then performed to evaluate the associations between physiological parameters, chief complaints, and PE at onset, and the diagnostic performance of identified warning factors was calculated.

**Results:**

A total of 5,120 patients who underwent TJA were evaluated. Symptomatic PE was diagnosed in 84 patients (1.64%), with a mean age of 67.4 ± 8.8 years. Among these, 73 underwent total knee arthroplasty (TKA) and 11 underwent total hip arthroplasty (THA). The mean time from surgery to PE onset was 51.8 ± 26.1 h, with 72 cases (85.7%) occurring within 72 h postoperatively. No significant differences in preoperative comorbidities were observed between the two groups (*P* > 0.05). At PE onset, pulse oxygen saturation (SpO_2_) monitoring revealed decreased SpO_2_ in 78 patients (92.9%). The most common presenting symptoms were respiratory manifestations (*n* = 48, 57.1%), followed by altered mental status (*n* = 22, 26.2%), precordial discomfort (*n* = 12, 14.3%), chills and fever (*n* = 8, 9.5%), fatigue (*n* = 7, 8.3%), and headache or dizziness (*n* = 4, 4.8%). Age-stratified analyses revealed significant differences in the distribution of SpO_2_, respiratory symptoms, and altered mental status (*P* < 0.05). Multivariable logistic regression identified lower levels of SpO_2_ (OR = 0.668, 95% CI: 0.599–0.745) as being independently associated with PE, while respiratory symptoms (OR = 16.448, 95% CI: 5.408–50.029) and altered mental status (OR = 5.828, 95% CI: 1.485–22.879) were established as independent predictors. The combined early recognition model incorporating these three parameters demonstrated an area under the receiver operating characteristic curve (AUC) of 0.966 (95% CI: 0.947–0.984), exhibiting superior warning performance compared to any single indicator.

**Conclusion:**

Post-TJA PE predominantly occurs within 72 h postoperatively. Decreased SpO_2_, respiratory symptoms, and altered mental status serve as early warning signs, with a combined early recognition model demonstrating superior warning performance compared to any single parameter. Early postoperative vital sign monitoring and recognition of age-related atypical symptoms should be enhanced to improve the early identification and management of PE.

## Introduction

Total joint arthroplasty (TJA) is one of the most common and successful orthopedic procedures worldwide. Over the past decade, the incidence of total hip arthroplasty (THA) and total knee arthroplasty (TKA) has increased by 130% and 150%, respectively (1). By 2050, global THA volumes will rise by 121% to over 200%, with TKA volumes increasing by at least 130% (1). This surge is primarily driven by population aging, rising obesity rates, expanding surgical indications to younger patients, and the growing adoption of TJA in developing regions (1–3). Despite the increasing volume of TJA procedures, ongoing surveillance of postoperative complications remains limited (2).

Pulmonary embolism (PE) is a potentially devastating complication following TJA. Reported incidence rates of post-TJA PE range from 0.19% to 3.4% (4, 5), with mortality rates from 0.08% to 4.33%, and the trend appears to be rising (3, 4). Moreover, beyond its severe clinical consequences—such as death, iatrogenic hemorrhage, post-thrombotic syndrome, and heparin-induced thrombocytopenia (4)—the economic and social burden of PE is also substantial. Consequently, PE remains a growing concern for surgeons, patients, and their families. Thus, the importance of proper recognition and accurate early diagnosis cannot be overemphasized.

In fact, the clinical presentation of PE is highly complex and lacks specificity (3, 6, 7). Symptoms can range from chest tightness, shortness of breath, hypoxemia, chest pain, and tachycardia to hemodynamic instability, altered mental status, or even sudden death in severe cases; occasionally, fever may be the sole manifestation (8). However, these initial symptoms frequently overlap with expected postoperative pain, anemia, or cardiopulmonary dysfunction, leading to missed or delayed diagnoses. Despite the availability of numerous diagnostic guidelines and predictive models, early identification of PE following TJA remains poor in real-world clinical practice (9). This is due to the atypical presentation of PE, which may even mask its clinical features (6, 8). Therefore, when patients present with atypical symptoms, the gold standard for confirming PE remains CT pulmonary angiography (CTPA) (3, 10). Furthermore, existing literature indicates that symptomatic PE typically occurs within 2–3 days postoperatively (3, 11). Hence, early recognition, accurate diagnosis, and timely management are of paramount importance.

Routine prophylactic anticoagulation has become the standard of care following TJA to prevent venous thromboembolism (VTE), including deep vein thrombosis (DVT) and PE. However, this preventive measure is hindered by the associated burdens of bleeding and infection (12). Moreover, PE may still occur regardless of prophylaxis (5, 13). Consequently, a critical challenge lies in accurately identifying patients at high risk for PE who require targeted prevention. Such risk stratification is essential to optimize resource allocation, enhance patient care, and minimize the incidence of life-threatening complications (14).

Given the current research landscape, this study retrospectively analyzed the onset time window and symptom spectrum of PE following TJA. The aim is to provide an evidence-based foundation for the early identification and intervention of PE, as well as to optimize the clinical management of high-risk patients.

## Materials and methods

### Patient population

Following approval by the Institutional Review Board of our hospital (No. 202608), we reviewed the discharge records of all patients who underwent TJA between January 2023 and December 2025. Patients with CTPA-confirmed postoperative PE were identified as the case group, and non-PE patients during the same period were randomly selected from the electronic medical record system as controls at a 1:2 ratio using computer-generated random sampling. Furthermore, multivariable logistic regression was performed to adjust for potential confounding factors, including age, BMI, duration of surgery, sex, and key comorbidities. Baseline characteristics, clinical features, and outcomes were collected. Baseline characteristics included sex, age, BMI, admission diagnosis, surgical procedure, operative duration, and preoperative comorbidities. Clinical features encompassed time from surgery to PE onset, the initial symptoms and SpO_2_ recorded at the time of suspected PE onset (i.e., when clinical suspicion first arose), and lower extremity venous ultrasonography findings. Outcomes were categorized as death, ongoing medication, and cure. Additionally, patients were stratified into three age groups: <60 years, 60–69 years, and ≥70 years. Furthermore, as this study primarily aimed to investigate the physiological and symptomatic changes at the exact moment of PE onset, no additional inclusion or exclusion criteria were applied.

At our institution, SpO_2_, vital signs, and clinical symptoms are routinely monitored in all patients following TJA. Therefore, data for all monitoring parameters were collected in both groups using identical protocols at comparable time points during the postoperative period. And SpO_2_ was measured via a standard finger pulse oximeter while patients received supplemental oxygen (nasal cannula at 1–2 L/min) per our routine postoperative protocol. Measurements were taken approximately every 3–4 h during the first 72 postoperative hours, and only the lowest SpO_2_ value was included in this study. Additionally, CTPA was performed to confirm the diagnosis in patients with suspected PE.

### Perioperative management

After routine preoperative workup and exclusion of contraindications, all procedures were performed by a senior arthroplasty surgeon. TKA was performed via an anterior midline approach using cemented prostheses, while THA was performed via a posterolateral approach using uncemented prostheses. Following implantation, a local analgesic-anesthetic cocktail was injected before wound closure. On the day of surgery, patients were mobilized under the supervision of a rehabilitation physician after complete recovery from anesthesia. During hospitalization, patients received standard analgesic and anticoagulant therapy. They also underwent physical therapy with a walker and a physical therapist to achieve an independent gait. Concurrently, comprehensive postoperative education was provided, with particular focus on resuming daily activities, preventing periprosthetic infection and dislocation, and managing osteoporosis. According to the anticoagulation guidelines for arthroplasty (15), our institutional regimen comprised routine anticoagulation for 10–14 days after TKA and for 4–5 weeks after THA. If lower extremity venous ultrasonography detected venous thromboembolism (VTE) of any degree during this period, the anticoagulation dosage was escalated and the treatment duration was extended.

Any perioperative symptoms or complaints were promptly managed with appropriate symptomatic treatment. For patients with suspected PE, CTPA and bedside lower extremity venous ultrasonography were performed. Once PE was confirmed, multidisciplinary team involvement was initiated immediately. The primary treatment strategy consisted of anticoagulation (low-molecular-weight heparin/LMWH, 1 IU/kg twice daily), oxygen supplementation, and supportive care. No patient received thrombolytic therapy. When determining the anticoagulation regimen, renal function was further evaluated, and informed consent for anticoagulant therapy was obtained. After 3 months of continuous anticoagulation, patients were reassessed every 2–3 weeks for PE symptoms, D-dimer levels, and lower extremity venous ultrasonography. If all parameters were normal, the anticoagulation regimen could be adjusted or discontinued.

### Statistical analysis

Statistical analyses were performed using SPSS 22.0 (IBM Corp., Armonk, NY, USA). Quantitative data with normal distribution are represented by mean ± standard deviation (SD), while categorical data are represented by frequencies and percentages (%). For normally distributed continuous variables, one-way repeated-measures ANOVA was used for intra-group comparisons, and independent-samples *t*-tests for inter-group comparisons. Categorical data were analyzed using the chi-square or Fisher's exact test, with Bonferroni correction for stratified comparisons. In addition, variables with *P* < 0.05 in the univariate analysis were considered for inclusion in the multivariable model. Correlation analysis and multivariable logistic regression were performed to identify independent predictors of PE, with results reported as odds ratios (ORs) and 95% confidence intervals (CIs). Multicollinearity was assessed using the variance inflation factor (VIF). Receiver operating characteristic (ROC) curve analysis was performed to evaluate the diagnostic performance of predictive factors. Given the limited sample size of the PE group, bootstrap resampling was employed for internal validation to assess model stability and potential overfitting. A two-sided *P* < 0.05 was considered statistically significant.

## Results

A total of 5,120 TJA patients (4,100 TKA and 1,020 THA) were reviewed, and 84 (1.64%) were diagnosed with symptomatic PE, including one perioperative death (1.19%). The PE cohort comprised 24 males and 60 females, with 73 (1.78%) after TKA and 11 (1.08%) after THA. Compared with the control group, patients in the PE group had comparable mean age (67.4 ± 8.8 vs. 67.0 ± 8.7 years), BMI (26.5 ± 3.9 vs. 27.0 ± 4.4 kg/m^2^), and operative time (75.6 ± 14.2 vs. 73.1 ± 13.4 min). The mean time to postoperative PE onset was 51.8 ± 26.1 h, with 15 (17.9%), 50 (59.5%), and 72 (85.7%) cases occurring within 24, 48, and 72 h, respectively. The most common initial presentation of PE was respiratory symptoms (chest tightness, shortness of breath, and dyspnea) in 48 cases (57.1%), followed by altered mental status (26.2%), precordial discomfort (palpitations, chest pain, and arrhythmias; 14.3%), chills and fever (9.5%), fatigue (8.3%), and headache or dizziness (4.8%). Notably, SpO_2_ monitoring showed that 78 patients (92.9%) had varying degrees of hypoxemia at symptom onset; among them, 5 elderly patients presented solely with decreased SpO_2_ and 3 solely with hypotension, all of whom were identified during routine vital sign monitoring. The mean lowest SpO_2_ at onset was 76.8% ± 11.1%. Compared with the non-PE group, the PE group showed significantly lower nadir SpO_2_ and higher incidences of respiratory symptoms and altered mental status (*P* < 0.05, Table 1). Concurrently with CTPA, lower extremity venous ultrasonography identified venous thrombosis of varying severity in 35 patients in the PE group; however, this showed no significant difference compared to routine postoperative follow-up findings in the non-PE group (*P* > 0.05).

**Table 1:** Patient baseline characteristics and postoperative abnormalities.

Variable	PE (*n* = 84)	Non-PE (*n* = 168)	*P*
Age (years)	67.4 ± 8.8	67.0 ± 8.7	0.796
BMI (kg/m^2^)	26.5 ± 3.9	27.0 ± 4.4	0.349
Operative time (min)	74.6 ± 14.2	73.1 ± 13.4	0.611
Lowest SpO_2_	76.8 ± 11.1	89.5 ± 3.8	**<0**.**001**
Respiratory symptoms	48	26	**<0**.**001**
Altered mental status	22	10	**<0**.**001**
Precordial discomfort	12	26	0.803
Chills/fever	8	20	0.571
Fatigue	7	15	0.875
Headache/dizziness	4	10	0.923
Preoperative LEVT	6	9	0.572
Postoperative LEVT	32	45	0.066

LEVT, lower extremity vein thrombosis.

Bold values indicate statistical significance.

The preoperative comorbidity profile showed that hypertension was the most common, present in 44 cases (52.4%), followed by osteoporosis/osteopenia (14, 16.7%), coronary artery disease (11, 13.1%), cerebral infarction (10, 11.9%), diabetes mellitus (9, 10.7%), and lower extremity deep vein thrombosis (6, 7.1%). Fifteen patients (17.8%) had no documented comorbidities. No significant differences in the prevalence of these comorbidities were observed between the PE and non-PE groups (*P* > 0.05).

Stratification by age revealed 14 patients (16.7%) aged <60 years, 34 (40.5%) aged 60–69 years, and 36 (42.9%) aged ≥70 years. Regarding initial presentations, significant age-related differences were observed in hypoxemia (71.4%, 94.1%, and 100%; *P* = 0.004), respiratory symptoms (85.7%, 67.6%, and 36.1%; *P* = 0.002), and altered mental status (0%, 23.5%, and 38.9%; *P* = 0.017) across the three respective age groups (Figure 1). However, no significant differences were found among the groups in precordial discomfort (14.3%, 11.7%, and 16.7%; *P* = 0.841), chills and fever (0%, 14.7%, and 8.3%; *P* = 0.151), fatigue (7.1%, 5.9%, and 11.1%; *P* = 0.721), or headache and dizziness (7.1%, 2.9%, and 5.6%; *P* = 0.784).

**Figure 1:**
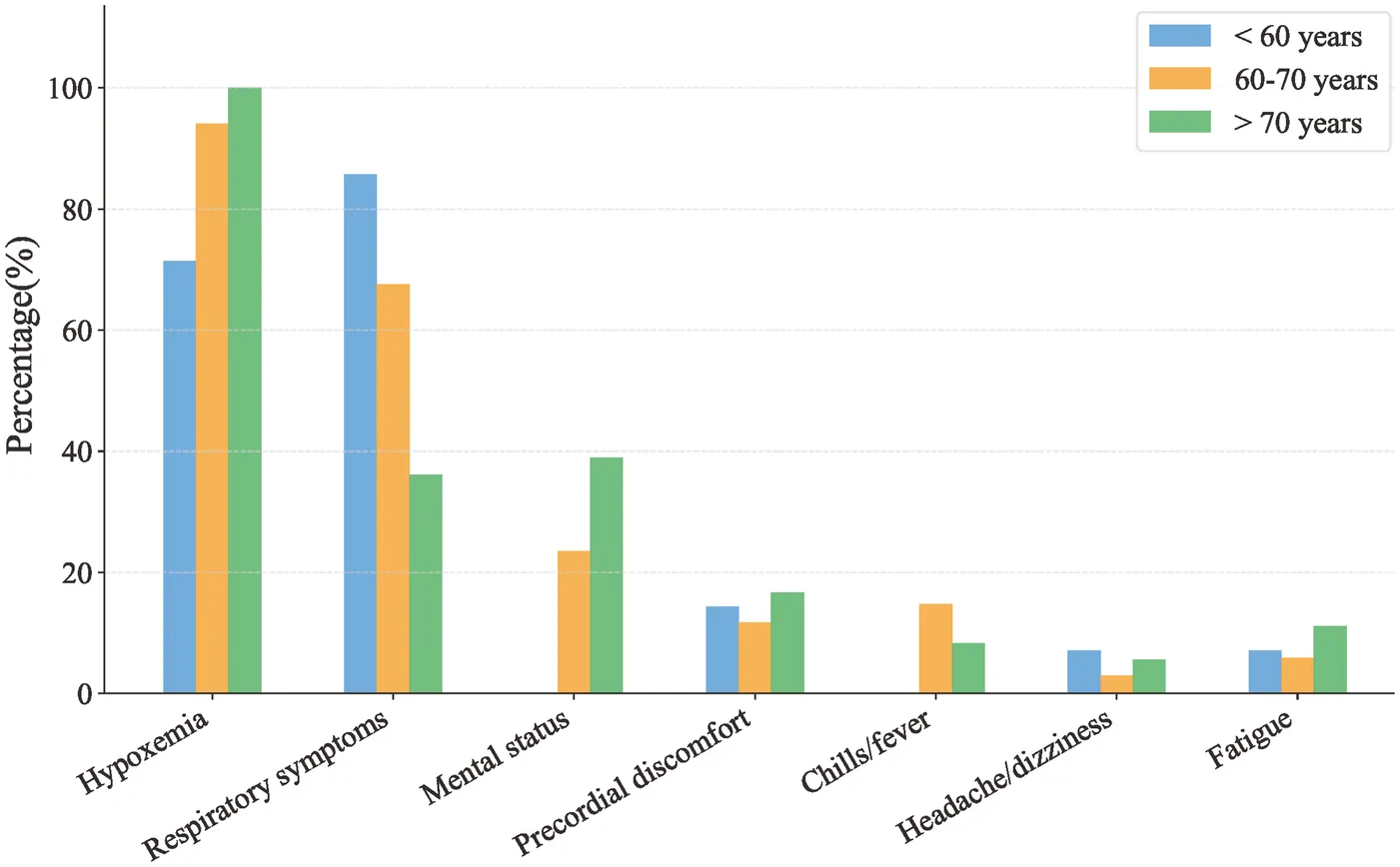
Proportion of initial presentation across age strata.

Univariate logistic regression analysis was performed to evaluate the impact of each variable on PE occurrence. The results indicated that lowest SpO_2_ (OR = 0.676, 95% CI: 0.617–0.741, *P* < 0.001), respiratory symptoms (OR = 7.282, 95% CI: 3.991–13.286, *P* < 0.001), and altered mental status (OR = 5.606, 95% CI: 2.511–12.517, *P* < 0.001) were significantly associated with PE. Conversely, age, BMI, operative time, sex, and comorbidities showed no statistically significant differences (*P* > 0.05) (Table 2).

**Table 2:** Univariate logistic regression analysis of warning factors of PE.

Relevant factors	OR (95% CI)	*B*	Wald	*P*
Gender	1.000 (0.560−1.786)	0.000	0.000	1.000
Age	1.004 (0.974–1.034)	0.004	0.067	0.795
BMI	0.970 (0.911–1.033)	−0.030	0.881	0.348
Operative time	1.005 (0.986–1.025)	0.005	0.261	0.610
Lowest SpO_2_	0.676 (0.617–0.741)	−0.392	**70**.**532**	**<0**.**001**
Surgical approach	1.000 (0.460–2.174)	0.000	0.000	1.000
Respiratory symptoms	7.282 (3.991–13.286)	1.985	**41**.**884**	**<0**.**001**
Altered mental status	5.606 (2.511–12.517)	1.724	**17**.**699**	**<0**.**001**
Precordial discomfort	0.910 (0.434–1.909)	−0.094	0.062	0.803
Chills/fever	0.779 (0.328–1.851)	−0.250	0.320	0.572
Fatigue	0.927 (0.363–2.369)	−0.076	0.025	0.875
Headache/dizziness	0.790 (0.240–2.598)	−0.236	0.151	0.698
Preoperative LEVT	1.359 (0.467–3.954)	0.307	0.317	0.573
Postoperative LEVT	1.682 (0.963–2.937)	0.520	3.345	0.067

LEVT, lower extremity vein thrombosis.

Bold values indicate statistical significance.

To adjust for confounders, variables with clinical significance in the univariate analysis were entered into a multivariate logistic regression model. The model passed the Omnibus test (*P* < 0.001), and the Hosmer–Lemeshow goodness-of-fit test showed satisfactory calibration (*P* = 0.359). Ultimately, lowest SpO_2_ (OR = 0.668, 95% CI: 0.599–0.745, *P* < 0.001), respiratory symptoms (OR = 16.448, 95% CI: 5.408–50.029, *P* < 0.001), and altered mental status (OR = 5.828, 95% CI: 1.485–22.879, *P* < 0.001) were identified as independent predictors of PE (Table 3). The VIF values for the three variables in the final model were: lowest SpO_2_ = 1.17, respiratory symptoms = 1.08, and altered mental status = 1.11, all well below the threshold of 5.0, indicating no concerning multicollinearity.

**Table 3:** Multivariate logistic regression analysis of warning factors of PE.

Relevant factors	OR (95% CI)	*B*	Wald	VIF	*P*
Lowest SpO_2_	0.668 (0.599, 0.745)	−0.403	52.397	1.17	**<0**.**001**
Respiratory symptoms	16.448 (5.408, 50.029)	2.800	24.344	1.08	**<0**.**001**
Altered mental status	5.828 (1.485, 22.879)	1.763	6.382	1.11	**<0**.**001**

Omnibus Tests (*P* < 0.000).

Hosmer–Lemeshow test (*P* = 0.359).

Bold values indicate statistical significance.

To evaluate the early recognition performance of individual warning factors and the combined model, ROC curves were generated. SpO_2_ demonstrated excellent diagnostic value (AUC = 0.923, 95% CI: 0.884–0.963, *P* < 0.001), with an optimal cutoff of 84.5% yielding a sensitivity of 0.905 and specificity of 0.893 based on the Youden index. The AUCs for respiratory symptoms and altered mental status were 0.708 (95% CI: 0.636–0.780, *P* < 0.001) and 0.601 (95% CI: 0.524–0.679, *P* = 0.009), respectively. Furthermore, a combined model incorporating the predicted probabilities from the multivariate regression achieved an AUC of 0.966 (95% CI: 0.947–0.984, *P* < 0.001). This significantly outperformed each individual warning factor, indicating robust early recognition efficacy for PE (Figure 2). The regression equation is Logit (P) = −4.405 + [−0.403 × (Lowest SpO_2_-84.5)] + (2.800 × Respiratory symptoms) + (1.763 × Altered mental status), where *P* is the probability of PE.

**Figure 2:**
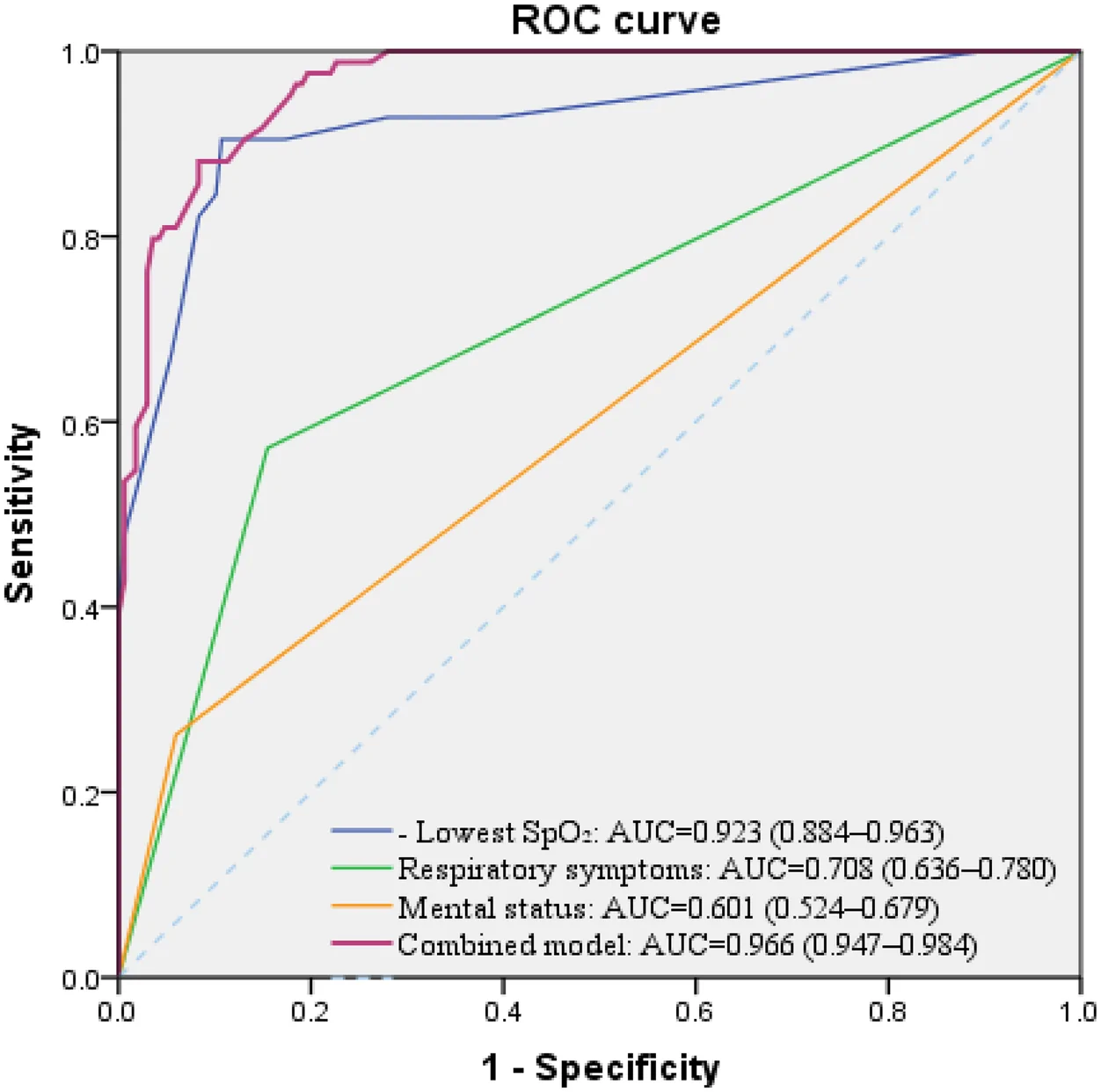
ROC curves for warning factors.

To further assess model stability and potential overfitting, internal validation was performed using bootstrap resampling (1,000 iterations). The bootstrap-corrected AUC was 0.923 (95% CI: 0.892–0.955), with an optimism of only 0.043 and a shrinkage factor of 0.955. These findings indicate robust performance and minimal overfitting despite the limited sample size (Figure 3).

**Figure 3:**
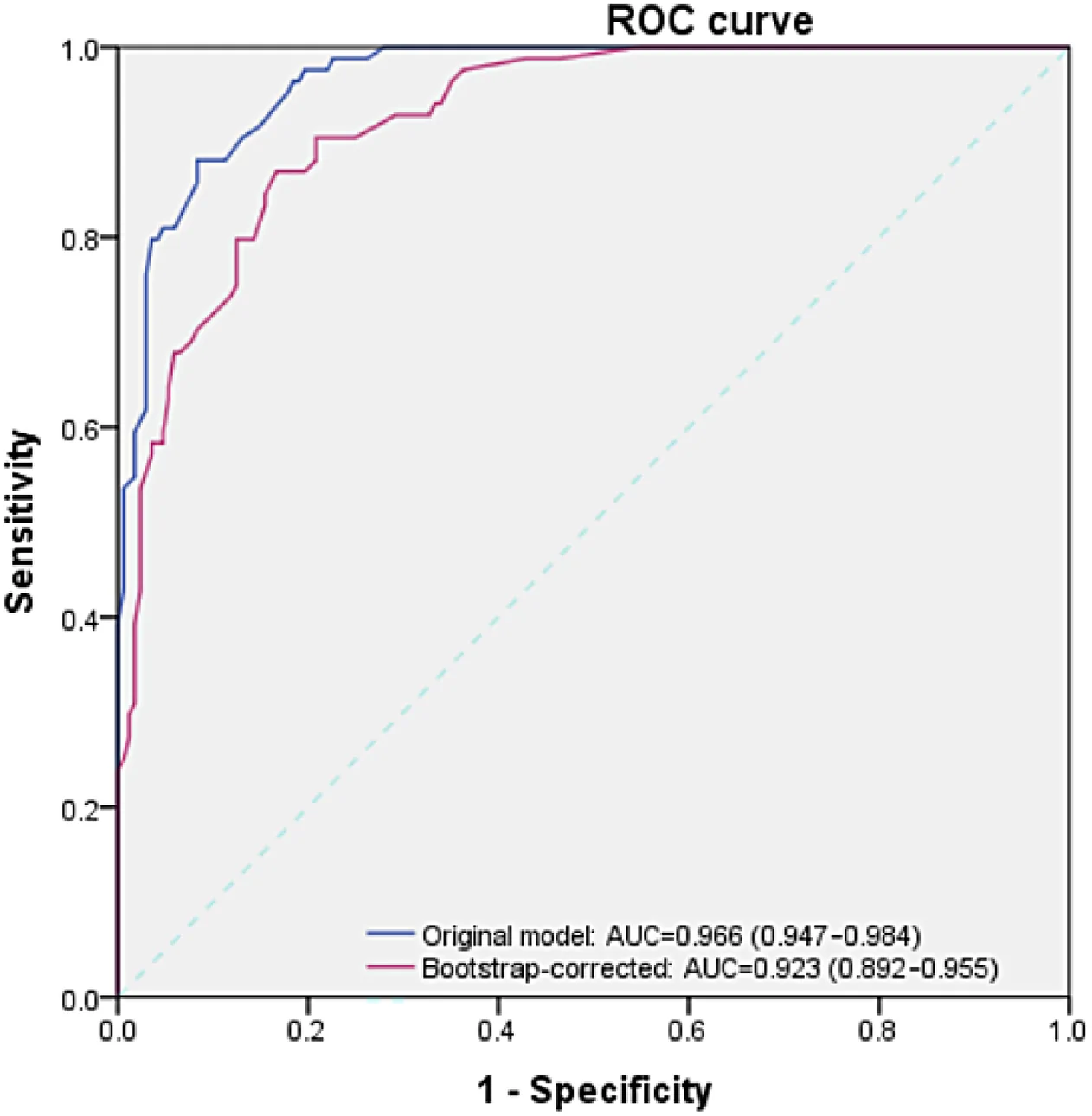
ROC curve for bootstrap-corrected model performance.

## Discussion

Based on the clinical data of 84 patients with PE onset after TJA, this retrospective study evaluated patient demographic characteristics, the onset time window, initial symptoms, and preoperative comorbidities. The overall incidence of symptomatic PE was 1.64%, with a mean onset time of 51.8 ± 26.1 h. Despite heterogeneous presenting symptoms, the independent predictors were primarily decreased SpO_2_, respiratory symptoms, and altered mental status. Therefore, integrating the onset time window with these independent predictors facilitates the early identification and management of post-TJA PE.

Numerous studies have investigated risk factors for PE following TJA, yet few have specifically focused on monitoring parameters and patient-reported symptoms at the exact moment of onset. The present study provides a predictive approach based on non-invasive findings and patient-reported symptoms at the time of PE occurrence, and thus does not incorporate preoperative laboratory variables. The primary aim is to guide clinicians toward more accurate empirical treatment at the first sign of physiological or symptomatic changes, rather than allowing delays in therapy while awaiting confirmatory investigations, such as CTPA.

Although symptomatic PE is relatively uncommon following TJA, its potentially fatal nature makes it a major concern for both clinicians and patients. The PE rate in our study was 1.64%, which is consistent with previous reports (3, 4). Nevertheless, this figure may still be underestimated, as asymptomatic cases were not included (16). Furthermore, post-TJA PE may not always be thrombotic; a substantial proportion may be attributable to bone cement. A cadaveric study of cemented prostheses detected PE in 46.3% of cases, suggesting that although the incidence of PE increases with cement use, it does not necessarily contribute to mortality (16). This finding further supports the higher PE risk associated with TKA compared with THA (3), a pattern also observed in our study.

In our study, the mean time to PE onset following TJA was 51.8 ± 26.1 h, with 85.7% of cases occurring within 72 h postoperatively, which is consistent with previous reports (3, 17, 18). However, initial clinical presentations were highly variable, and not all patients exhibited classic PE symptoms such as chest pain, hemoptysis, and dyspnea. Notably, we observed age-related differences in symptom profiles: hypoxemia, hypotension, and altered mental status were more prevalent in older patients, whereas respiratory symptoms were more common in relatively younger cohorts. Such age-dependent variations have been sporadically reported in the literature (19–21). We attribute these differences to three main factors. First, older patients typically have reduced pulmonary compliance, impaired cough reflexes, and lower ventilatory reserves. Consequently, they are more susceptible to postoperative atelectasis, secretion retention, aspiration, exacerbated sleep apnea, or mild heart failure, all of which can lead to decreased SpO_2_. Even under identical stress levels, elderly patients are therefore more prone to early hypoxemia. Second, the aging brain exhibits heightened sensitivity to hypoxia, hypoperfusion, and systemic inflammation. Third, symptom communication is often compromised in older adults; subjective complaints of chest tightness or shortness of breath may be atypical or absent. Instead, they frequently manifest as agitation, lethargy, disorientation, uncooperativeness, or delayed responses, clinically mimicking primary altered mental status. Based on these findings, our team emphasizes that relying solely on patient-reported symptoms for early PE recognition in elderly TJA patients significantly increases the risk of missed diagnoses. Therefore, accurate and timely identification of post-TJA PE remains critically important.

Regarding risk prediction for PE following TJA, previous studies have identified advanced age, cardiovascular diseases, diabetes, obesity, and a history of thrombosis as risk factors for post-TJA PE (3, 14, 22–24). However, in our cohort, preoperative comorbidities did not differ significantly between PE patients and the general TJA population during the same period. This discrepancy may be attributed to the low proportion of patients with a BMI ≥ 40 kg/m^2^, a narrower spectrum of comorbidities, or potential selection bias in the control group. Furthermore, our institution routinely conducts multidisciplinary preoperative consultations for high-risk patients (e.g., those with cardiac disease or DVT). Interventions, including targeted treatments or even surgical postponement, effectively reduce the pool of high-risk patients undergoing surgery. Notably, preoperative venous thromboembolism in our cohort was primarily confined to the calf muscle veins. In this study, univariate and multivariate binary logistic regression analyses identified decreased SpO_2_, respiratory symptoms, and altered mental status as independent warning factors of PE. Based on these variables, we constructed a combined early recognition model. ROC analysis demonstrated that the combined predictive performance was significantly superior to that of any single indicator, suggesting high clinical value for the early identification of PE. Furthermore, Youden index analysis based on SpO_2_ values indicated an optimal cutoff of 84.5%, yielding a sensitivity of 0.905 and a specificity of 0.893. Indeed, although pre- and postoperative lower extremity venous thrombosis are not independent risk factors for PE in our cohort, they still pose a substantial risk for PE development (trending toward significance). Therefore, new-onset postoperative lower extremity venous thrombosis warrants close monitoring.

Notably, all three independent predictors in our ROC model demonstrated excellent diagnostic performance. Specifically, SpO_2_ yielded an AUC of 0.923 (95% CI: 0.884–0.963), respiratory symptoms an AUC of 0.708 (95% CI: 0.636–0.780), and altered mental status an AUC of 0.601 (95% CI: 0.524–0.679). Furthermore, the combined model performed even better, achieving an AUC of 0.966 (95% CI: 0.947–0.984), which outperformed previously reported predictive models (23, 25). We attribute this enhanced performance to the temporal focus of our study. Unlike previous studies (9, 23) that relied on preoperative comorbidities and laboratory results to identify risk factors, our model evaluates clinical parameters at the exact moment of acute PE onset. Indeed, the predominant contribution of SpO_2_ to the model's accuracy can be explained by our clinical protocol. Preoperatively, patients with resting SpO_2_ below 85% undergo multidisciplinary evaluation and are rarely admitted for surgery. Postoperatively, all TJA patients receive continuous intermittent low-flow nasal cannula oxygen (1–2 L/min). Therefore, despite supplemental oxygen, a decline in SpO_2_ serves as a critical and highly specific warning sign, reflecting the underlying pathophysiological impairment of gas exchange caused by PE.

This study primarily focuses on readily available bedside parameters (SpO_2_) and patient-reported symptoms at the onset of PE. The resulting early recognition model requires no complex diagnostics and may facilitate early recognition of PE in similar clinical settings, suggesting potential clinical utility, though external validation is needed before widespread implementation. Importantly, routine anticoagulation guidelines for TJA do not support blind prophylactic high-dose anticoagulation in preoperatively identified high-risk patients, considering both economic costs and bleeding risks, given that all TJA patients are classified as very high risk for postoperative thrombosis by the Caprini score (23). Furthermore, despite prophylaxis, some individuals will inevitably develop PE (5, 13). Therefore, only through early recognition at symptom onset and effective individualized intervention can the population-level cost-effectiveness (in terms of both expenditure and bleeding risk) far outweigh that of immediate high-dose postoperative prophylactic anticoagulation.

This study has several limitations. First, it is a single-center retrospective analysis, and the sample size and population characteristics may be subject to selection bias. Thus, our findings warrant further validation through multicenter, prospective studies. Second, the relatively narrow range of body habitus and comorbidity profiles due to ethnic homogeneity limits the generalizability of our results, and future investigations with larger, more diverse populations are warranted. Third, although bootstrap internal validation of the early recognition model has demonstrated its robustness, further validation in larger, multicenter external cohorts is required to confirm its generalizability. Fourth, this study did not assess right ventricular dysfunction, troponin levels, echocardiographic findings, or the Pulmonary Embolism Severity Index (PESI) at the onset of PE. Future prospective studies are warranted to incorporate standardized severity scoring systems and comprehensive hemodynamic assessments to further elucidate the dynamic changes of PE following TJA.

## Conclusion

Post-TJA PE predominantly occurs within the 72 h postoperatively. Decreased SpO_2_, respiratory symptoms, and altered mental status serve as early warning signals, with a combined early recognition model demonstrating superior diagnostic efficacy compared to any single indicator. Notably, clinical presentations at onset vary by age, with older patients being more susceptible to hypoxemia and altered mental status. Therefore, enhanced early postoperative vital signs monitoring and the recognition of age-related atypical symptoms are crucial to facilitate the early identification and timely intervention of PE.

## Data Availability

The original contributions presented in the study are included in the article/Supplementary Material, further inquiries can be directed to the corresponding author.

## References

[1] Stubnya BG Schulz M Váncsa S Szilágyi GS Szatmári A Bejek Z. Global trends in joint arthroplasty: a systematic review and future projections. JCM. (2025) 14:8214. 10.3390/jcm14228214 41303249 PMC12653837

[2] Siddiqi A Jacob P Yousuf KM. Beyond case counts: defining quality and accountability in short-term arthroplasty missions. J Bone Joint Surg Am. (2026) 108:1047–50. 10.2106/JBJS.26.00279 41973830

[3] Arnindita JN Mahyuddin MH Saputra PBT Putra RM. Identifying pulmonary embolism - high risk patients after total knee replacement: systematic review. J Pak Med Assoc. (2024) 74:S41–50. 10.47391/JPMA.S6-ACSA-08 39018138

[4] Reddy GB Ovadia JE Yakkanti RR Browne JA D'Apuzzo RM. Increased morbidity with diagnosis and treatment of pulmonary embolism after total joint arthroplasty: a matched control analysis of 30,000 patients. J Arthroplasty. (2022) 37:948–52. 10.1016/j.arth.2022.01.086 35143922

[5] Lieberman JR Cheng V Cote MP. Pulmonary embolism rates following total hip arthroplasty with prophylactic anticoagulation: some pulmonary emboli cannot be avoided. J Arthroplasty. (2017) 32:980–6. 10.1016/j.arth.2016.09.006 27866948

[6] Chai Z Hu R Ma C. Acute pulmonary embolism with loss of consciousness as the first manifestation: a case report. Ann Transl Med. (2023) 11:304. 10.21037/atm-23-656 37181339 PMC10170283

[7] Chen B Wang W Zang W Hu J Li H Wang C. Analysis of clinical characteristics in 1940 patients with acute pulmonary embolism. Respir Med. (2025) 237:107920. 10.1016/j.rmed.2024.107920 39725333

[8] Natour HH Elgardt I Rotman-Pikielny P Natour A Natour A Levy Y. Masquerading pulmonary embolism: fever as an overlooked clinical manifestation illustrated by four mini case series and literature review. Am J Med. (2025) 138:1124–7. 10.1016/j.amjmed.2025.02.026 40043866

[9] Ding R Ding Y Zheng D Huang X Dai J Jia H. Machine learning-based screening of risk factors and prediction of deep vein thrombosis and pulmonary embolism after hip arthroplasty. Clin Appl Thromb Hemost. (2023) 29:10760296231186145. 10.1177/10760296231186145 37394825 PMC10328010

[10] Meyer G Becattini C Geersing G-J Harjola V-P Huisman MV Humbert M. 2019 ESC guidelines for the diagnosis and management of acute pulmonary embolism developed in collaboration with the European Respiratory Society (ERS). Eur Heart J. (2020) 41(4):543–603. 10.1093/eurheartj/ehz405 31504429

[11] Hu C Liu C Wang Y Ding T Sun K Tian S. The timing of symptomatic pulmonary embolism in patients with nonwarfarin anticoagulants following elective primary total joint arthroplasty. J Arthroplasty. (2020) 35:1703–7. 10.1016/j.arth.2020.01.024 32046872

[12] Azboy I Barrack R Thomas AM Haddad FS Parvizi J. Aspirin and the prevention of venous thromboembolism following total joint arthroplasty: commonly asked questions. Bone Joint J. (2017) 99-B(11):1420–30. 10.1302/0301-620X.99B11.BJJ-2017-0337.R2 29092979 PMC5742873

[13] Cote MP Chen A Jiang Y Cheng V Lieberman JR. Persistent pulmonary embolism rates following total knee arthroplasty even with prophylactic anticoagulants. J Arthroplasty. (2017) 32:3833–9. 10.1016/j.arth.2017.06.041 28807470

[14] Bulut H Maestre MJ Tomey D. Predictors and risk factors of pulmonary embolism after total hip arthroplasty: an NSQIP study. Hip Intl. (2025) 35:70–5. 10.1177/11207000241270205 39171673

[15] Anderson DR Morgano GP Bennett C Dentali F Francis CW Garcia DA. American Society of Hematology 2019 guidelines for management of venous thromboembolism: prevention of venous thromboembolism in surgical hospitalized patients. Blood Adv. (2019) 3:3898–944. 10.1182/bloodadvances.2019000975 31794602 PMC6963238

[16] Ritter J Hubert J Kniep I Beil FT Rolvien T Püschel K. Pulmonary cement embolism is frequently observed but not a contributing factor for death in patients with cemented total hip and knee arthroplasty: a postmortem study. Int Orthop. (2022) 46:1225–32. 10.1007/s00264-022-05381-6 35352160 PMC9117385

[17] Samama C-M Ravaud P Parent F Barré J Mertl P Mismetti P. Epidemiology of venous thromboembolism after lower limb arthroplasty: the FOTO study. J Thromb Haemost. (2007) 5:2360–7. 10.1111/j.1538-7836.2007.02779.x 17908282

[18] Senay A Trottier M Delisle J Banica A Benoit B Laflamme GY. Incidence of symptomatic venous thromboembolism in 2372 knee and hip replacement patients after discharge: data from a thromboprophylaxis registry in Montreal, Canada. VHRM. (2018) 14:81–9. 10.2147/VHRM.S150474 PMC5951148 29780248

[19] Sapota-Zaręba K Nasierowski T. Pulmonary embolism masked by symptoms of mental disorders. Postep Psychiatr Neurol. (2023) 32:147–51. 10.5114/ppn.2023.132118 PMC10683049 38034502

[20] Almalki KA Idris KJ Alzahrani AA Ahmed S. “Complication unveiled: bilateral pulmonary embolism in the wake of shoulder arthroplasty” a case report. Int J Surg Case Rep. (2025) 129:111148. 10.1016/j.ijscr.2025.111148 40120327 PMC11981809

[21] Ahaneku CA Akpu BB Njoku CH Elem DE Ekeng BE. Pulmonary embolism presenting as delirium: an acute confusional state in an elderly patient—a case report. Egypt J Intern Med. (2023) 35:8. 10.1186/s43162-023-00193-5 36777903 PMC9899661

[22] Thompson R Cassidy R Hill J Bryce L Napier R Beverland D. Are patients with morbid obesity at increased risk of pulmonary embolism or proximal deep vein thrombosis after lower limb arthroplasty? A large-database study. Clin Orthop Relat Res. (2024) 482:115–24. 10.1097/CORR.0000000000002742 37404124 PMC10723888

[23] Liu M Zhou Z Ma X Ma J Wu X Chen B. Multivariate logistic regression analysis to explore the high-risk factors of deep vein embolism/pulmonary embolism after knee replacement in the elderly. J Orthop Surg (Hong Kong). (2025) 33:10225536251345202. 10.1177/10225536251345202 40424507

[24] Kusnezov N Iyer A Richardson MK Piple AS Wang JC Liu KC. Risk factors for venous thromboembolism following total knee arthroplasty: an analysis of 3,052 pulmonary emboli. J Am Acad Orthop Surg. (2025) 33:e380–90. 10.5435/JAAOS-D-23-00426 39657170

[25] Liu KC Bagrodia N Richardson MK Piple AS Kusnezov N Wang JC. Risk factors associated with thromboembolic complications after total hip arthroplasty: an analysis of 1,129 pulmonary emboli. J Am Acad Orthop Surg. (2024) 32:e706–15. 10.5435/JAAOS-D-23-01213 38626438

